# 3-[211At]astato-4-fluorobenzylguanidine: a potential therapeutic agent with prolonged retention by neuroblastoma cells.

**DOI:** 10.1038/bjc.1997.366

**Published:** 1997

**Authors:** G. Vaidyanathan, X. G. Zhao, R. H. Larsen, M. R. Zalutsky

**Affiliations:** Department of Radiology, Duke University Medical Center, Durham, NC 27710, USA.

## Abstract

An analogue of meta-iodobenzylguanidine (MIBG) in which an aromatic hydrogen was replaced with fluorine has been found to possess many properties similar to those of the parent compound. Moreover, 4-fluoro-3-iodobenzylguanidine (FIBG) was retained in vitro by human neuroblastoma cells to a much greater extent than MIBG itself. Since alpha-emitters such as 211At could be valuable for the treatment of micrometastatic disease, an FIBG analogue in which the iodine atom is replaced by 211At would be of interest. In this study, we have evaluated the in vitro and in vivo properties of 3-[211At]astato-4-fluorobenzylguanidine ([211At]AFBG). The specific binding of [211At]AFBG to SK-N-SH human neuroblastoma cells remained fairly constant over 2- to 3-log activity range and was similar to that of [131I]MIBG. The uptake of [211At]AFBG by this cell line was reduced by desipramine, ouabain, 4 degrees C incubation, noradrenaline, unlabelled MIBG and FIBG, suggesting that its uptake is specifically mediated through an active uptake-1 mechanism. Over the 16 h period studied, the amount of [211At]AFBG retained was similar to that of [131I]FIBG, whereas the per cent of retained meta-[211At]astatobenzylguanidine ([211At]MABG) was considerably less than that of [131I]FIBG (53% vs 75%; P < 0.05). The IC50 values for the inhibition of uptake of [131I]MIBG, [211At]MABG, [125I]FIBG and [211At]AFBG by unlabelled MIBG were 209, 300, 407 and 661 nM respectively, suggesting that the affinities of these tracers for the noradrenaline transporter in SK-N-SH cells increase in that order. Compared with [211At]MABG, higher uptake of [211At]AFBG was seen in vivo in normal mouse target tissues such as heart and, to a certain extent, in adrenals. That the uptake of [211At]AFBG in these tissues was related to the uptake-1 mechanism was demonstrated by its reduction when mice were pretreated with desipramine. However, the stability of [211At]AFBG towards in vivo dehalogenation was less than that of [211At]MABG, as evidenced by the higher uptake of 211At in thyroid, spleen, lungs and stomach.


British Journal of Cancer (1997) 76(2), 226–233
© 1997 Cancer Research Campaign

### 3-[$^{211}$At]astato-4-fluorobenzylguanidine: a potential therapeutic agent with prolonged retention by neuroblastoma cells

**G Vaidyanathan, X-G Zhao, RH Larsen and MR Zalutsky**

Department of Radiology, Duke University Medical Center, Durham, NC 27710, USA

**Summary** An analogue of *meta*-iodobenzylguanidine (MIBG) in which an aromatic hydrogen was replaced with fluorine has been found to possess many properties similar to those of the parent compound. Moreover, 4-fluoro-3-iodobenzylguanidine (FIBG) was retained in vitro by human neuroblastoma cells to a much greater extent than MIBG itself. Since α-emitters such as $^{211}$At could be valuable for the treatment of micrometastatic disease, an FIBG analogue in which the iodine atom is replaced by $^{211}$At would be of interest. In this study, we have evaluated the in vitro and in vivo properties of 3-[$^{211}$At]astato-4-fluorobenzylguanidine ([$^{211}$At]AFBG). The specific binding of [$^{211}$At]AFBG to SK-N-SH human neuroblastoma cells remained fairly constant over 2- to 3-log activity range and was similar to that of [$^{131}$I]MIBG. The uptake of [$^{211}$At]AFBG by this cell line was reduced by desipramine, ouabain, 4°C incubation, noradrenaline, unlabelled MIBG and FIBG, suggesting that its uptake is specifically mediated through an active uptake-1 mechanism. Over the 16 h period studied, the amount of [$^{211}$At]AFBG retained was similar to that of [$^{131}$I]FIBG, whereas the per cent of retained *meta*-[$^{211}$At]astatobenzylguanidine ([$^{211}$At]MABG) was considerably less than that of [$^{131}$I]FIBG (53% vs 75%; $P < 0.05$). The $IC_{50}$ values for the inhibition of uptake of [$^{131}$I]MIBG, [$^{211}$At]MABG, [$^{125}$I]FIBG and [$^{211}$At]AFBG by unlabelled MIBG were 209, 300, 407 and 661 nM respectively, suggesting that the affinities of these tracers for the noradrenaline transporter in SK-N-SH cells increase in that order. Compared with [$^{211}$At]MABG, higher uptake of [$^{211}$At]AFBG was seen in vivo in normal mouse target tissues such as heart and, to a certain extent, in adrenals. That the uptake of [$^{211}$At]AFBG in these tissues was related to the uptake-1 mechanism was demonstrated by its reduction when mice were pretreated with desipramine. However, the stability of [$^{211}$At]AFBG towards in vivo dehalogenation was less than that of [$^{211}$At]MABG, as evidenced by the higher uptake of $^{211}$At in thyroid, spleen, lungs and stomach.

**Keywords:** neuroblastoma; *meta*-iodobenzylguanidine; 3-[$^{211}$At]astato-4- fluorobenzylguanidine

Iodine-131-labelled *meta*-iodobenzylguanidine ([$^{131}$I]MIBG) has been used for the targeted radiotherapy of a number of neuroendocrine tumours, such as neuroblastoma (Klingebiel et al, 1989; Garaventa et al, 1991). Although treatment of neuroblastoma with [$^{131}$I]MIBG has been shown to be efficacious, improvements in this therapeutic modality are needed and a number of approaches are under active investigation. For example, combination of [$^{131}$I]MIBG treatment with high-dose chemotherapy and total body irradiation is being pursued (Gaze et al, 1995). For micrometastases, the long-range β-particles of $^{131}$I are suboptimal (Sisson et al, 1990; O'Donoghue et al, 1991) and the development of an MIBG analogue labelled with an α-emitter such as 7.2 h half-life $^{211}$At has been advocated (Shapiro et al, 1987; Mairs et al, 1991).

We developed a method for the synthesis of *meta*-[$^{211}$At]astatobenzylguanidine ([$^{211}$At]MABG) and preliminary studies showed that this compound behaved in a similar fashion to MIBG (Vaidyanathan and Zalutsky, 1992; Vaidyanathan et al, 1994*a*). In clonogenic assays with the SK-N-SH human neuroblastoma cell line, the $D_0$ value for [$^{211}$At]MABG was 0.2 kBq ml$^{-1}$ compared with 384 kBq ml$^{-1}$ for no carrier added (n.c.a.) [$^{131}$I]MIBG, indicating a more than 1000-fold greater cytotoxicity for the α-particle-emitting analogue over [$^{131}$I]MIBG under single-cell conditions (Strickland et al, 1994).

Recently, a fluorine-containing analogue of MIBG was developed for positron emission tomography (Vaidyanathan et al, 1994*b*; 1995). This agent, 4-fluoro-3-iodobenzylguanidine (FIBG; Figure 1), also could be labelled with iodine radionuclides (Vaidyanathan et al, 1996). The uptake and retention of FIBG in SK-N-SH human neuroblastoma cells in vitro and mouse target tissues (heart and adrenals) were significantly higher than those of MIBG. Encouraged by the exquisite cytotoxicity delivered by [$^{211}$At]MABG, an astatinated analogue of FIBG also was synthesized (Vaidyanathan et al, 1996). Herein, we have evaluated this new agent, 3-[$^{211}$At]astato-4-fluorobenzylguanidine ([$^{211}$At]AFBG), with respect to its uptake and retention in SK-N-SH cells in vitro and tissue distribution in normal mice. Like [$^{131}$I]MIBG and [$^{131}$I]FIBG, [$^{211}$At]AFBG is taken up by SK-N-SH cells by an active uptake-1 mechanism. In comparison with [$^{211}$At]MABG, higher levels of [$^{211}$At]AFBG were retained in SK-N-SH cells in vitro and in normal, uptake-1-containing tissues (heart and adrenals) in mice.

*Received 30 August 1996*
*Revised 16 January 1997*
*Accepted 28 January 1997*

*Correspondence to:* Ganesan Vaidyanathan, Box 3808, Department of Radiology, Duke University Medical Center, Durham, NC 27710, USA

### MATERIALS AND METHODS

### General

All chemicals were purchased from Aldrich Chemical Company except as noted. Unlabelled MIBG, desipramine (DMI) and noradrenaline (arterenol; NE) were obtained from Sigma. Sodium

Figure 1 Chemical structure of [$^{131}$I]MIBG, [$^{131}$I]FIBG, [$^{18}$F]FIBG, [$^{211}$At]MABG and [$^{211}$At]AFBG

Figure 2 Paired-label dose-dependent binding of [$^{211}$I]AFBG and [$^{131}$I]MIBG to SK-N-SH human neuroblastoma cells in vitro

[$^{131}$I]iodide in 0.1 N sodium hydroxide was supplied by DuPont-New England Nuclear (North Billerica, MA, USA).

### Preparation of radiohalogenated benzylguanidines

*Meta*-iodobenzylguanidine (Vaidyanathan and Zalutsky, 1993) and 4-fluoro-3-iodobenzylguanidine (Vaidyanathan et al, 1996) were labelled with $^{131}$I at the no-carrier-added level, following procedures reported previously. Briefly, to the required amount of [$^{131}$I]iodide in 1–3 μl of 0.1 N sodium hydroxide was added 10 μl of 0.3 M solution of *N*-chlorosuccinimide in trifluoroacetic acid followed by the corresponding silicon precursor in trifluoroacetic acid (5 μl of 0.1 M solution). After 5 min at room temperature, the product was isolated by reversed-phase chromatography. The $^{211}$At activity was produced on the Duke University CS-30 cyclotron via the $^{209}$Bi(α, 2n)$^{211}$At reaction by bombarding natural bismuth metal targets with 28-MeV α-particles (Zalutsky et al, 1989; Larsen et al, 1996). The activity was isolated from the target by dry distillation into either 0.1 N sodium hydroxide (0.1 ml) or chloroform (0.5 ml). The activity trapped in 0.1 N sodium hydroxide was concentrated to less than 3 μl, or the activity trapped in chloroform was extracted in more than 95% efficiency into 3 μl of 0.1 N sodium hydroxide for the preparation of astatinated benzylguanidines. To the activity obtained as above in a Reacti vial was added 10 μl of *N*-chlorosuccinimide and the corresponding precursor solutions in trifluoroacetic acid, as described for the iodination reactions. The reaction mixture was heated at 70°C for 5 min and 12 min, for [$^{211}$At]MABG (Vaidyanathan and Zalutsky, 1992) and [$^{211}$At]AFBG (Vaidyanathan et al, 1996) preparation respectively, and the products were isolated by reversed-phase chromatography.

No co-eluting mass peak was detected in the UV trace of HPLC analyses of any of these radiolabelled benzylguanidines. To date, a maximum of at least 37 MBq of each tracer has been produced. Since the detection limit of iodobenzylguanidines is about 1 nmol, the specific activities of radiohalogenated benzylguanidines is expected to be greater than 37 TBq mmol$^{-1}$. As there are no stable isotopes of astatine, a specific activity nearer the theoretical limit (16 000 TBq mmol$^{-1}$) is more likely for the astatinated agents.

### Measurement of radioactivity

A dose calibrator (Capintec, CRC-7R, USA) for higher amounts of activity and an automated gamma counter (LKB 1282, Wallac, Finland) for lower count rates were used. For measurement of $^{211}$At activity using the gamma-counter, the energy window was set to encompass the Po K X-rays emitted by its electron capture decay branch. In the dose calibrator, the window was set to that of $^{133}$Xe. Both gamma-counter and dose calibrator had been cross-calibrated for $^{211}$At with a germanium semiconductor detector. For paired-label studies, gamma-counter counting windows were set to a dual-channel to encompass the 77–92 keV Po X-rays emitted by $^{211}$At and the 364-keV gamma-rays of $^{131}$I. Counting data were automatically corrected for the 11% crossover of $^{131}$I in the $^{211}$At gate and the physical decay of both nuclides.

### Cells and culture conditions

The human neuroblastoma cell lines SK-N-SH (uptake-1 positive) and SK-N-MC (uptake-1 negative) (Biedler et al, 1973) were purchased from American Type Culture Collection (Rockville, MD, USA). The incubation medium (JRH Biosciences, Lenexa, KS, USA) was made by mixing 440 ml of RPMI-1640, 50 ml of Serum Plus, 5 ml of penicillin-G/streptomycin (5000 U of penicillin and 5000 μg of streptomycin in 1 ml) and 5 ml of glutamine (200 mM in saline). The cells were grown at 37°C in a humidified incubator containing 5% carbon dioxide. Cell viability was evaluated before each binding experiment by trypan blue dye and was 95–98% for all studies.

© Cancer Research Campaign 1997

**Figure 3** Effect of various agents and conditions on the uptake of [211At]AFBG by SK-N-SH human neuroblastoma cells

**Figure 4** Retention of benzylguanidines by SK-N-SH cells after removal of unbound activity in the presence and absence of DMI. **(A)** [131I]FIBG and [211At]AFBG., ⊙, [211At]AFBG; ▣, [211At]AFBG+DMI; ●, [131I]FIBG; ■, [131I]FIBG+DMI. **(B)** [131I]FIBG and [211At]MABG, ●, [211At]MABG; ■, [211At]MABG+DMI; ◎, [131I]FIBG; ▣, [131I]FIBG+DMI

### Paired-label in vitro binding of [211At]AFBG and [131I]MIBG to SK-N-SH human neuroblastoma cells

The cells were seeded into 24-well plates (4 × $10^5$ cells in 500 μl medium per well) and incubated for 24 h in a 37°C/5% carbon dioxide humidified atmosphere. After removing the medium, fresh medium containing 0.26–50.32 kBq of [131I]MIBG and [211At]AFBG was added and the cells were incubated for 2 h at 37°C. The medium was removed at the end of incubation and the cells were washed twice with phosphate-buffered saline. The cells were solubilized by incubation with 500 μl of 0.5 N sodium hydroxide for 30 min at room temperature and then removed with cotton swabs. The cell-bound activity was counted along with input standards using a dual-channel gamma-counter. Each measurement was performed in quadruplicate. In all cases, non-specific binding was determined by preincubating cells with DMI (50 μM) for 30 min before adding the tracers, or using the uptake-1 negative cell line SK-N-MC.

### Specificity of [211At]AFBG uptake in SK-N-SH cells

The cells were seeded into 24-well plates (4 × $10^5$ cells in 500 μl of medium per well) and incubated for 24 h. To determine the effect of DMI and ouabain, after removing medium the cells were pre-incubated with 500 μl of medium containing 1.5 μM DMI or 1 mM ouabain for 30 min at 37°C. After this period, the medium was aspirated and fresh medium containing 2.8 kBq of [211At]AFBG was added. Cells were then incubated for 2 h at 37°C. To determine the effect of temperature, cells were plated as above. After 24 h, medium was removed, 450 μl of fresh medium was added and incubated at 4°C for at least 1 h. Subsequently, 2.8 kBq of [211At]AFBG was added followed by incubation for 2 h at 4°C. Blocking effects of NE (50 μM), MIBG (10 μM) and FIBG (10 μM) were determined by co-incubating the cells with the tracers and these agents for 2 h at 37°C. In all experiments, the cell-associated activity was determined as described above. The per cent binding to the cells was normalized to incubations performed simultaneously without interventional agents. Each measurement was performed in quadruplicate.

### Paired-label retention of [211At]AFBG and [131I]FIBG, and [131I]FIBG and [211At]MABG, in SK-N-SH cells

Cells were loaded onto 24-well plates at a density of 5 × $10^5$ cells per well per 500 μl of medium and incubated at 37°C for 24 h. After the 24-h period, the medium was replaced with fresh medium containing 5.6 kBq of each tracer in a total volume of 500 μl per well. The cells were incubated with the activity for 2 h at 37°C. At the end of this incubation period, the medium was removed and supplemented with 500 μl of fresh medium with and without 1.5 μM DMI. The cell-bound activity was determined at 0, 2, 4, 6, 16 and 24 h after the initial uptake. Each time point was done in quadruplicate. The activity retained at each time point was normalized to the initially bound activity to calculate the per cent retained activity.

© Cancer Research Campaign 1997

**Figure 5** Effect of graded concentrations of unlabelled MIBG on the uptake of [$^{131}$I]MIBG, [$^{125}$I]FIBG, [$^{211}$At]MABG and [$^{211}$At]AFBG by SK-N-SH cells. ○, [$^{131}$I]MIBG; □, [$^{211}$At]MABG; ◇, [$^{125}$I]FIBG; △, [$^{211}$At]AFBG

### Effect of graded concentrations of unlabelled MIBG on the uptake of [$^{131}$I]MIBG, [$^{125}$I]FIBG, [$^{211}$At]MABG and [$^{211}$At]AFBG by SK-N-SH cells

Cells ($4 \times 10^5$) were incubated in quadruplicate with 3.7 kBq of each tracer in the absence and presence of various concentrations (10 pM to 10 μM) of unlabelled MIBG at 37°C for 2 h and the percentage cell-bound activity was determined as above. The specific binding at each concentration of MIBG was obtained by subtracting the binding observed at 10 μM MIBG from the binding at each concentration. These values were normalized to the maximum binding and plotted against the MIBG concentrations.

### Biodistribution in normal mice

Several experiments were performed. Male BALB/c mice weighing about 25 g were used for all experiments. Groups of five mice were used for each time point. The tracers were injected via the tail vein. The mice were killed by an overdose of halothane and tissues of interest were isolated, washed, blot-dried and counted for $^{131}$I and $^{211}$At using a dual-label programme in a gamma-counter. A 5% or 10% aliquot of the injected activity was also counted so that the percentage of injected dose per organ or per gram of tissue could be computed.

Tissue uptake of [$^{211}$At]AFBG was compared with that of [$^{131}$I]MIBG at 1 h and 4 h after injection and with that of [$^{131}$I]FIBG at 1 h, 4 h and 24 h by administering 185–370 kBq of each tracer. The tissue distribution of [$^{211}$At]MABG and [$^{211}$At]AFBG at 1 h, 4 h and 24 h was also determined in parallel experiments. The specificity of uptake was determined by investigating the effect of desipramine on the uptake of [$^{211}$At]AFBG in the heart and adrenals, tissues that have an active uptake-1 mechanism. In these experiments mice were pretreated with a PBS solution of desipramine (10 mg $kg^{-1}$) i.p. 30 min before administering the tracers. The control group received just the saline vehicle and the tracer. The tissue uptake was determined at 1 h after injection of the radioactivity. Statistical significance of differences was determined by paired or independent Student *t*-tests. The differences were considered to be significant if *P*-values were less than 0.05.

### RESULTS

### Radioactivity-dependent binding of [$^{131}$I]MIBG and [$^{211}$At]AFBG to SK-N-SH cells

The specific binding of [$^{131}$I]MIBG and [$^{211}$At]AFBG by SK-N-SH cells as a function of activity concentration is shown in Figure 2. The binding of [$^{211}$At]AFBG was reasonably constant (40–61%) in the activity range studied, and was virtually identical to that of no-carrier-added [$^{131}$I]MIBG at each activity level. The non-specific binding, determined by pretreatment of SK-N-SH cells with DMI and/or by using SK-N-MC line, was less than 2% in all cases.

### Specificity of uptake by SK-N-SH cells

The uptake of [$^{211}$At]AFBG was blocked, to varying degrees, by several interventional agents and incubation at 4°C (Figure 3). At a concentration of 1.5 μM, the uptake-1 inhibitor DMI reduced the uptake of [$^{211}$At]AFBG to 17% of the control values. Whereas ouabain (1 mM) reduced the uptake to 37% of control values, incubation at 4°C reduced it to 11%. These data suggest that the uptake process is energy dependent. The specificity of uptake is further demonstrated by its inhibition by 50 μM noradrenaline, 10 μM MIBG and 10 μM FIBG to 9%, 7% and 6%, respectively, of control values.

### Retention of [$^{211}$At]AFBG and [$^{211}$At]MABG by SK-N-SH cells

The ability of SK-N-SH cells to retain [$^{211}$At]AFBG and [$^{211}$At]MABG in comparison with that of [$^{131}$I]FIBG is shown in Figure 4A and B. Over the 16-h period studied, the amount of [$^{211}$At]AFBG retained was similar to that of [$^{131}$I]FIBG. In contrast, by 16 h, the amount of initially bound [$^{211}$At]MABG was considerably less than that of [$^{131}$I]FIBG (53% vs 75%; $P < 0.05$). The difference was even more apparent at 24 h (42% vs 73%; $P < 0.05$). Treatment with DMI diminished the capacity of the cells to retain [$^{211}$At]AFBG and [$^{211}$At]MABG, suggesting that reuptake of these tracers in SK-N-SH cells occurs as is seen with MIBG.

### $IC_{50}$ of MIBG for blocking the uptake of [$^{131}$I]MIBG, [$^{125}$I]FIBG, [$^{211}$At]MABG and [$^{211}$At]AFBG by SK-N-SH cells

Figure 5 shows the inhibition of uptake of various benzylguanidines by graded concentrations of unlabelled MIBG. The pattern of the curves is similar for all four benzylguanidines. The $IC_{50}$ values were 209, 300, 407 and 661 nM respectively for [$^{131}$I]MIBG, [$^{211}$At]MABG, [$^{125}$I]FIBG and [$^{211}$At]AFBG, suggesting that the affinities for these tracers to the noradrenaline transporter in SK-N-SH cells increase in that order.

### Biodistribution studies

Tables 1 and 2 show the data from biodistributions of [$^{211}$At]AFBG in comparison with that of [$^{131}$I]MIBG and [$^{131}$I]FIBG. At 1 h, the heart uptake of [$^{211}$At]AFBG (22.4 ± 2.1% ID/g) was not different

© Cancer Research Campaign 1997

**Table 1** Paired-label tissue distribution of [$^{211}$At]AFBG and [$^{131}$I]MIBG in BALB/c mice

| Tissue | Per cent injected dose per gram of tissue[a] | | | |
|---|---|---|---|---|
| | 1 h | | 4 h | |
| | [$^{211}$At]AFBG | [$^{131}$I]MIBG | [$^{211}$At]AFBG | [$^{131}$I]MIBG |
| Liver | 6.1 ± 1.0 | 8.5 ± 0.7 | 2.6 ± 0.3 | 4.5 ± 0.6 |
| Spleen | 7.0 ± 0.7 | 4.6 ± 0.8 | 5.3 ± 0.7 | 3.5 ± 0.5 |
| Lungs | 14.4 ± 4.7 | 9.3 ± 2.9 | 5.6 ± 1.3 | 3.9 ± 0.7 |
| Heart | 22.4 ± 2.1 | 22.7 ± 3.0[b] | 18.4 ± 2.2 | 14.8 ± 1.6 |
| Kidney | 4.2 ± 0.4 | 3.0 ± 0.4 | 3.1 ± 0.3 | 1.9 ± 0.2 |
| Stomach | 12.0 ± 4.8 | 4.8 ± 1.8 | 11.4 ± 1.7 | 2.0 ± 0.3 |
| Small intestine | 4.9 ± 0.6 | 8.2 ± 0.9 | 2.3 ± 0.5 | 3.8 ± 0.7 |
| Large intestine | 3.1 ± 0.4 | 3.8 ± 0.6 | 2.0 ± 0.2 | 4.6 ± 0.6 |
| Thyroid[c] | 0.7 ± 0.1 | 0.5 ± 0.1 | 1.6 ± 0.5 | 0.5 ± 0.3 |
| Muscle | 2.3 ± 0.3 | 2.1 ± 0.3 | 1.3 ± 0.2 | 1.2 ± 0.2 |
| Bone | 1.4 ± 0.2 | 1.1 ± 0.2 | 1.0 ± 0.2 | 0.8 ± 0.2 |
| Blood | 1.5 ± 0.1 | 0.9 ± 0.1 | 0.9 ± 0.1 | 0.4 ± 0.0 |
| Brain | 0.2 ± 0.0 | 0.1 ± 0.0 | 0.2 ± 0.1 | 0.1 ± 0.1 |
| Adrenals | 18.8 ± 1.6 | 19.1 ± 2.5[b] | 22.0 ± 8.4 | 26.2 ± 9.7 |

[a]Mean ± s.d.; five animals per time point. [b]Differences not statistically significant ($P > 0.05$) by paired *t*-test. [c]Per cent injected dose per organ.

**Table 2** Paired-label tissue distribution of [$^{211}$At]AFBG and [$^{131}$I]FIBG in BALB/c mice

| Tissue | Per cent injected dose per gram of tissue[a] | | | | | |
|---|---|---|---|---|---|---|
| | 1 h | | 4 h | | 24 h | |
| | [$^{211}$At]AFBG | [$^{131}$I]FIBG | [$^{211}$At]AFBG | [$^{131}$I]FIBG | [$^{211}$At]AFBG | [$^{131}$I]FIBG |
| Liver | 4.7 ± 1.1 | 7.9 ± 1.2 | 2.7 ± 0.2 | 7.2 ± 0.3 | 0.7 ± 0.1 | 1.9 ± 0.2 |
| Spleen | 5.9 ± 0.5 | 4.0 ± 0.2 | 5.1 ± 0.4 | 3.1 ± 0.3 | 2.6 ± 0.6 | 3.5 ± 0.7 |
| Lungs | 9.9 ± 1.8 | 9.0 ± 1.2 | 5.4 ± 1.0 | 4.8 ± 0.4[b] | 2.2 ± 0.6 | 1.8 ± 0.4[b] |
| Heart | 22.1 ± 4.9 | 21.4 ± 5.2 | 17.4 ± 1.6 | 18.1 ± 2.0 | 7.8 ± 1.6 | 10.9 ± 2.0 |
| Kidney | 3.3 ± 0.3 | 2.6 ± 0.3 | 2.7 ± 0.3 | 2.0 ± 0.2 | 1.3 ± 0.2 | 1.2 ± 0.2[b] |
| Stomach | 6.4 ± 2.0 | 2.2 ± 1.0 | 11.3 ± 3.8 | 2.6 ± 1.3 | 6.5 ± 1.3 | 1.3 ± 0.2 |
| Small intestine | 3.1 ± 0.8 | 4.8 ± 1.3 | 2.9 ± 0.2 | 4.0 ± 0.3 | 1.3 ± 0.2 | 2.1 ± 0.0 |
| Large intestine | 2.6 ± 0.5 | 3.2 ± 0.7 | 2.6 ± 0.4 | 4.1 ± 0.5 | 1.0 ± 0.1 | 2.0 ± 0.2 |
| Thyroid[c] | 0.8 ± 0.3 | 0.6 ± 0.2 | 1.7 ± 1.0 | 0.7 ± 0.5 | 1.0 ± 0.2 | 0.3 ± 0.1 |
| Muscle | 2.1 ± 0.2 | 1.7 ± 0.2 | 1.8 ± 0.2 | 1.4 ± 0.2 | 0.5 ± 0.1 | 0.7 ± 0.1 |
| Bone | 1.3 ± 0.2 | 0.9 ± 0.1 | 1.3 ± 0.1 | 0.8 ± 0.1 | 0.3 ± 0.1 | 0.2 ± 0.0[b] |
| Blood | 1.4 ± 0.1 | 0.9 ± 0.0 | 1.1 ± 0.1 | 0.5 ± 0.1 | 0.3 ± 0.1 | 0.1 ± 0.0 |
| Brain | 0.3 ± 0.1 | 0.2 ± 0.1 | 0.5 ± 0.3 | 0.3 ± 0.4 | 0.1 ± 0.0 | 0.1 ± 0.1[b] |
| Adrenals | 16.0 ± 3.0 | 15.3 ± 3.1[b] | 14.8 ± 3.8 | 13.2 ± 2.1[b] | 8.6 ± 1.5 | 12.5 ± 0.9 |

[a]Mean ± s.d. ($n = 5$). [b]Differences not statistically significant ($P > 0.05$) by a paired *t*-test. [c]Per cent injected dose per organ.

($P > 0.05$) from that of [$^{131}$I]MIBG (22.7 ± 3.0% ID/g). However, at 4 h, the heart uptake of [$^{211}$At]AFBG (18.4 ± 2.2% ID/g) was about 25% higher ($P < 0.05$) than that of [$^{131}$I]MIBG (14.8 ± 1.6% ID/g). At 1 h and 4 h, the heart uptake of [$^{211}$At]AFBG (22.1 ± 4.9% ID/g and 17.4 ± 1.6% ID/g respectively) and [$^{131}$I]FIBG (21.4 ± 5.2 and 18.1 ± 2.0% ID/g respectively) was similar; at 24 h, the heart uptake of [$^{131}$I]FIBG (10.9 ± 2.0% ID/g) was significantly higher than that of [$^{211}$At]AFBG (7.8 ± 1.6% ID/g). The adrenal uptake of [$^{211}$At]AFBG, while similar at early time points to that of the iodinated benzylguanidines, was significantly lower at later time points. The specificity of uptake of [$^{211}$At]AFBG in vivo was demonstrated by its inhibition with DMI. One hour after the tracer injection, DMI pretreatment reduced the heart and adrenal uptake of [$^{211}$At]AFBG to 68% and 71% of the control values respectively ($P < 0.05$). Compared with [$^{131}$I]MIBG, spleen, lungs and stomach showed significantly higher uptake of [$^{211}$At]AFBG. Lung and spleen uptake of [$^{211}$At]AFBG was similar to that of [$^{131}$I]FIBG; however, stomach uptake of [$^{211}$At]AFBG was three- to fivefold higher than that of [$^{131}$I]FIBG. Thyroid uptake of [$^{211}$At]AFBG was two- to threefold higher than that of [$^{131}$I]MIBG and [$^{131}$I]FIBG. Liver and intestines retained [$^{211}$At]AFBG to a lower degree than [$^{131}$I]MIBG and [$^{131}$I]FIBG. As shown in Table 3, results from parallel experiments showed that spleen, lung, thyroid and stomach uptake of [$^{211}$At]AFBG was consistently higher than that of [$^{211}$At]MABG, suggesting that the lesser stability of the former towards dehalogenation in vivo.

### DISCUSSION

An important factor in the optimization of endoradiotherapy is to match the properties of the emitted radiation with the characteristics of the tumour target. For micrometastatic disease, the radionuclide

 © Cancer Research Campaign 1997

**Table 3** Tissue distribution of [$^{211}$At]AFBG and [$^{211}$At]MABG in BALB/c mice

| Tissue | Per cent injected dose per gram of tissue[a] | | | | | |
|---|---|---|---|---|---|---|
| | 1 h | | 4 h | | 24 h | |
| | [$^{211}$At]AFBG | [$^{211}$At]MABG | [$^{211}$At]AFBG | [$^{211}$At]MABG | [$^{211}$At]AFBG | [$^{211}$At]MABG |
| Liver | 6.2 ± 1.0 | 10.6 ± 1.2 | 3.3 ± 0.5 | 5.2 ± 0.7 | 1.0 ± 0.2 | 1.5 ± 0.2 |
| Spleen | 7.0 ± 1.0 | 5.5 ± 0.1 | 6.2 ± 0.7 | 4.9 ± 0.3 | 2.8 ± 0.3 | 3.4 ± 0.5 |
| Lungs | 14.5 ± 3.2 | 10.7 ± 1.4 | 6.7 ± 1.8 | 5.2 ± 0.9[b] | 2.5 ± 0.4 | 2.2 ± 0.4[b] |
| Heart | 22.6 ± 1.6 | 26.7 ± 1.4 | 19.5 ± 2.7 | 17.1 ± 1.3[b] | 9.7 ± 0.8 | 7.3 ± 0.6 |
| Kidney | 3.8 ± 0.4 | 3.6 ± 0.3[b] | 3.5 ± 1.0 | 2.4 ± 0.1 | 1.5 ± 0.2 | 1.5 ± 0.3[b] |
| Stomach | 11.6 ± 4.2 | 5.7 ± 1.3[b] | 13.3 ± 4.1 | 5.4 ± 0.7 | 4.4 ± 1.4 | 3.7 ± 0.8[b] |
| Small intestine | 4.7 ± 0.8 | 7.5 ± 0.8 | 3.2 ± 0.5 | 5.4 ± 0.8 | 1.2 ± 0.2 | 2.8 ± 0.5 |
| Large intestine | 3.0 ± 0.6 | 3.6 ± 0.5[b] | 2.4 ± 0.4 | 5.1 ± 0.6 | 0.9 ± 0.1 | 2.2 ± 0.3 |
| Thyroid[c] | 0.6 ± 0.1 | 0.4 ± 0.1 | 1.4 ± 0.5 | 0.5 ± 0.2 | 0.5 ± 0.1 | 0.3 ± 0.1[b] |
| Muscle | 2.6 ± 0.4 | 3.0 ± 0.2[b] | 1.7 ± 0.2 | 1.9 ± 0.1 | 0.6 ± 0.1 | 0.8 ± 0.1 |
| Bone | 1.7 ± 0.4 | 1.5 ± 0.2[b] | 1.6 ± 0.0 | 1.2 ± 0.4[b] | 0.2 ± 0.1 | 0.4 ± 0.1 |
| Blood | 1.3 ± 0.1 | 0.9 ± 0.1 | 0.9 ± 0.2 | 0.4 ± 0.1 | 0.2 ± 0.0 | 0.2 ± 0.1[b] |
| Brain | 0.2 ± 0.0 | 0.2 ± 0.0 | 0.2 ± 0.0 | 0.1 ± 0.1 | 0.1 ± 0.0 | 0.0 ± 0.0[b] |
| Adrenals | 13.6 ± 0.9[d] | 21.6 ± 0.7 | 13.4 ± 2.3 | 12.3 ± 3.0[b] | 11.4 ± 4.0 | 15.0 ± 2.6[b] |

[a]Mean ± s.d. ($n$ = 5). [b]Differences not statistically significant ($P$ > 0.05) by a Student $t$-test. [c]Per cent injected dose per organ. [d]$n$ = 4.

$^{211}$At is particularly appealing since its α-particles deposit their energy over a range equivalent to only a few cell diameters. In addition, these α-particles are radiations of high linear energy transfer with a relative biological effectiveness higher than β-particles, such as those emitted during the decay of $^{131}$I. This has led a number of investigators to suggest that a $^{211}$At-labelled analogue of [$^{131}$I]MIBG might be a more effective agent for the treatment of neuroblastoma (Shapiro and Gross, 1987; Kemshead et al, 1990; Mairs et al, 1991). Indeed, recent in vitro experiments have confirmed that under single cell conditions, the cytotoxicity of [$^{211}$At]MABG was three orders of magnitude higher than that of [$^{131}$I]MIBG (Strickland et al, 1994).

The results obtained in vitro with [$^{211}$At]MABG have been highly encouraging; however, other $^{211}$At-labelled MIBG analogues may have even more favourable properties as endoradiotherapeutic agents. While rapid uptake of [$^{211}$At]MABG in human neuroblastoma cell lines was achieved, rapid washout was also observed, particularly in the SK-N-SH line, in which a retention half-life of 5–6 h was seen (Strickland et al, 1994). Recently, we have reported that substitution of a fluorine atom *ortho* to the iodine in MIBG resulted in an MIBG analogue, [$^{131}$I]FIBG, with modestly increased binding, but significantly enhanced retention of radioiodine in SK-N-SH cells in vitro (Vaidyanathan et al, 1997). Given the similar behaviour of [$^{211}$At]MABG and [$^{131}$I]MIBG, we performed the current study to determine whether fluorine substitution *ortho* to the astatine atom in [$^{211}$At]MABG would have a similar effect.

As a paired-label comparison of [$^{211}$At]MABG and [$^{211}$At]AFBG would not be possible because of the lack of appropriate astatine radionuclide for use in tandem with $^{211}$At, two separate comparisons were performed. The first demonstrated that the uptake and retention of [$^{211}$At]AFBG by SK-N-SH cells was essentially identical to that of [$^{131}$I]FIBG over 16 h, an experimental period over which the $^{211}$At had decayed to about 21% of initial levels. In the second paired-label experiment, the binding of [$^{211}$At]MABG was compared directly with that of [$^{131}$I]FIBG. Unlike the results of the previous experiment, the maximal uptake of $^{211}$At activity was significantly lower than that of $^{131}$I. In addition, the retention of [$^{211}$At]MABG by this neuroblastoma cell line was lower than that of [$^{131}$I]FIBG, with the difference increasing with time. These results suggest that the radiotoxicity of [$^{211}$At]AFBG for SK-N-SH cells should be even greater than that of [$^{211}$At]MABG and experiments are planned to confirm this speculation.

If [$^{211}$At]AFBG is to be pursued as an endoradiotherapeutic agent, it is critical to understand the mechanisms responsible for its uptake not only in human tumour cells, but also in normal tissues such as heart and adrenals, where uptake-1-mediated localization could be problematical. In SK-N-SH cells, MIBG is taken up by a neuron-specific active uptake-1 mechanism (Buck et al, 1985; Smets et al, 1989), a process that has been demonstrated to occur with the analogues [$^{211}$At]MABG (Vaidyanathan and Zalutsky, 1994*a*), [$^{18}$F]FIBG (Vaidyanathan et al, 1994*b*, 1995) and [$^{131}$I]FIBG Vaidyanathan et al, 1997).

The results of this study suggest that the uptake-1 mechanism is also responsible for the accumulation of [$^{211}$At]AFBG in SK-N-SH neuroblastoma cells. The tricyclic antidepressant DMI, norepinephrine, MIBG and FIBG all were effective in blocking uptake of [$^{211}$At]AFBG to a similar degree as observed for [$^{131/125}$I]MIBG, [$^{211}$At]MABG and [$^{18}$F/$^{131}$I]FIBG. In addition, [$^{211}$At]AFBG accumulation is energy dependent as performing the incubation at 4°C, or in the presence of ouabain, resulted in a marked decrease in cell binding. Finally, uptake of [$^{211}$At]AFBG was reduced significantly in the heart and adrenals of mice pretreated with the uptake-1 inhibitor DMI, albeit to a slightly lesser degree than that reported for MIBG and other halobenzylguanidine analogues (Vaidyanathan et al, 1995; 1997; Valette et al, 1993). Taken together, these results suggest that substitution of astatine for iodine in the FIBG molecule does not interfere with the uptake-1 mechanism responsible for the accumulation of halobenzylguanidines in this human neuroblastoma cell line.

Differences in the mechanism of tracer uptake between [$^{211}$At]AFBG and [$^{211}$At]MABG thus do not explain the higher uptake and retention of the former in the SK-N-SH cell line. One possibility is that the presence of the fluorine atom enhances the inertness of [$^{211}$At]AFBG to catabolic breakdown, as fluorine substitution has been a general tactic to increase the metabolic

© Cancer Research Campaign 1997

stability of a variety of pharmaceuticals (Barnette, 1984). With regard to our particular application, as fluorine is the most electronegative element, its presence could stabilize weaker adjacent carbon–halogen bonds (Goldman, 1969). However, as discussed later, this is contradicted by the fact that deastatination of [$^{211}$At]AFBG appears to occur more readily than [$^{211}$At]MABG.

Substitution of fluorine for hydrogen in MIBG has been shown to increase slightly lipophilicity (Vaidyanathan et al, 1994*b*) and as a result, could partially account for the increased binding and retention of FIBG by SK-N-SH cells. If lipophilicity were the predominating factor, then one would expect [$^{211}$At]AFBG to exhibit even higher binding because of the greater lipophilicity of astatine compared with iodine (Vaidyanathan et al, 1994*b*); however, our results indicate nearly identical in vitro behaviour of [$^{211}$At]AFBG and [$^{131}$I]FIBG. Other possible effects of fluoro substitution on the cellular retention of halobenzylguanidine analogues, include alterations in the affinity for the norepinephrine transporter working in the reverse mode (Servidei et al, 1995) or changes in the intracellular storage site. The affinity of four halobenzylguanidines for the uptake transporter is indeed different, as shown by the $IC_{50}$ values of MIBG for their inhibition.

Decreasing the loss of label from an endoradiotherapeutic agent is important not only for maximizing radiation absorbed dose to tumour, but also for minimizing deleterious effects to normal tissues. MIBG is considered to be metabolically stable (Mangner et al, 1986); however, paired-label studies in normal mice have demonstrated that thyroid uptake of radioiodine following administration of [$^{131}$I]FIBG was 2–3 times lower than that from [$^{125}$I]MIBG, suggesting an enhanced inertness to deiodination for the former (Vaidyanathan et al, 1997). Based on these results, we anticipated that substitution of a fluorine atom *ortho* to the astatine would decrease the rate of deastatination as well.

The relative selectivity of astatide for spleen and lungs is about ten times higher than that for iodide (Garg et al, 1990), so accumulation of activity in these tissues following paired injection of $^{211}$At- and $^{131}$I-labelled analogues is consistent with deastatination in vivo. When the tissue distribution of [$^{211}$At]AFBG and [$^{131}$I]FIBG was compared, uptake of both radionuclides in the lungs was quite similar and no consistent trend was observed with regard to $^{211}$At/$^{131}$I spleen uptake ratios. However, the accumulation of [$^{211}$At]AFBG in the spleen, lungs, thyroid and, barring a few, in most other tissues was higher than that seen for [$^{211}$At]MABG. This could reflect a general effect of fluorine substitution, analogous to the increased tissue retention of FIBG compared with MIBG (Vaidyanathan et al, 1997), or a greater susceptibility of [$^{211}$At]AFBG to deastatination. Although the latter is a distinct possibility, it is difficult to explain why *ortho* substitution of a fluorine for a hydrogen could decrease the rate of deiodination but increase the rate of deastatination. Labelled catabolite analyses will be performed in order to investigate further the nature of these differences.

In summary, the results of this study demonstrate that the uptake characteristics of [$^{211}$At]AFBG in the SK-N-SH human neuroblastoma line are mediated by an active uptake-1 mechanism and, like other no-carrier-added halobenzylguanidine preparations, is not saturable over a 2-log activity range. Compared with [$^{211}$At]MABG, [$^{211}$At]AFBG offers the advantage of higher retention in neuroblastoma cells in vitro, but the disadvantage of higher retention in normal tissues in vivo. Preliminary results of studies performed in an athymic mouse human neuroblastoma xenograft model, to be published elsewhere, indicate tumour-to-normal tissue ratios for [$^{211}$At]AFBG in heart, adrenals and liver that are higher than those observed with [$^{211}$At]MABG.

### ACKNOWLEDGEMENTS

This work was supported in part by grants CA60066 and CA42324 from the National Institutes of Health. The authors wish to thank Donna J Affleck, Susan Slade and Phil Welsh for their excellent technical assistance.

### REFERENCES

Barnette WE (1984) The synthesis and biology of fluorinated prostacyclins. *CRC Critical Rev Biochem* **15**: 201–235

Biedler JN, Helson L and Spengler BA (1973) Morphology and growth, tumorigenicity, and cytogenetics of human neuroblastoma cells in continuous culture. *Cancer Res* **33**: 2643–2652

Buck J, Bruchelt G, Girgert R, Treuner J and Niethammer D (1985) Specific uptake of m-[$^{125}$I]iodobenzylguanidine in the human neuroblastoma cell line SK-N-SH. *Cancer Res* **45**: 6366–6370

Garaventa A, Guerra P, Arrighini A, Bertolazzi L, Bestagno M, De Bernardi B, Lanino E, Villavecchia GP and Claudiani F (1991) Treatment of advanced neuroblastoma with I-131 meta-iodobenzylguanidine. *Cancer* **67**: 922–928

Garg PK, Harrison CL and Zalutsky MR (1990) Comparative tissue distribution in mice of the α-emitter $^{211}$At and $^{131}$I as labels of a monoclonal antibody and F(ab`)$_2$ fragment. *Cancer Res* **50**: 3514–3520

Gaze MN, Wheldon TE, O'Donoghue JA, Hiditch TE, McNee SG, Simpson E and Barrett A (1995) Multi-modality megatherapy with [$^{131}$I]-meta-iodobenzylguanidine, high dose melphalan and total body irradiation with bone marrow rescue: Feasibility study of an innovative strategy for advanced neuroblastoma. *Eur J Cancer* **31A**: 252–256

Goldman P (1969) The carbon–fluorine bond in compounds of biological interest. *Science* **164**: 1123–1130

Kemshead JT, Pizer PL and Patel K (1990) Neuroblastoma: Perspectives for future research. In *Neuroblastoma: Tumor Biology and Therapy*. Pochedly C (ed.), pp 381–395. CRC Press: Boca Raton, FL

Klingebiel T, Treuner J, Ehninger G, Keller KD, Dopfer R, Feine U and Niethammer D (1989) [$^{131}$I]-Metaiodobenzylguanidine in the treatment of metastatic neuroblastoma. Clinical, pharmacological and dosimetric aspects. *Cancer Chemother Pharmacol* **25**: 143–148

Larsen RH, Wieland BW and Zalutsky MR (1996) Evaluation of an internal cyclotron target for the production of astatine-211 via the $^{209}$Bi(α,2n)$^{211}$At reaction. *Appl Radiat Isot* **47**: 135–143

Mangner TJ, Tobes MC, Wieland DW, Sisson JC and Shapiro B (1986) Metabolism of iodine-131 metaiodobenzylguanidine in patients with metastatic pheochromocytoma. *J Nucl Med* **27**: 37–44

Mairs R, Angerson WJ, Babich JW and Murray T (1991) Differential penetration of targeting agents into multicellular spheroids derived from human neuroblastoma. In *Advances in Neuroblastoma Research 3*, Prog. Clin. Biol. Res. Vol. 366, Evans AE, D'Angio GJ, Knudson Jr AG, and Seeger RC (eds), pp. 495–501. Wiley-Liss: New York

O'Donoghue A, Wheldon TE, Babich JW, Moyes JSE, Barret A and Meller T (1991) Implications of the uptake of $^{131}$I-radiolabelled meta-iodobenzylguanidine (MIBG) for the targeted radiotherapy of neuroblastoma. *Br J Radiol* **64**: 428–434

Servidei T, Iavarone A, Lasorella A, Mastrangelo S and Riccardi R (1995) Release mechanisms of [$^{125}$I]meta-iodobenzylguanidine in neuroblastoma cells: Evidence of a carrier-mediated efflux. *Eur J Cancer* **31A**: 591–595

Shapiro N and Gross MD (1987) Radiochemistry, biochemistry, and kinetics of $^{131}$I-metaiodobenzylguanidine (MIBG) and $^{123}$I-MIBG: Clinical implications of the use of $^{123}$I-MIBG. *Med Pediatr Oncol* **15**: 170–177

Sisson JC, Hutchinson RJ, Shapiro B, Zasadny KR, Normolle D, Wieland DM, Wahl RL, Singer DA, Mallete SA and Mudgett EE (1990) Iodine-125-MIBG to treat neuroblastoma: Preliminary report. *J Nucl Med* **31**: 1479–1485

Smets LA, Loesberg C, Janssen M, Metwally EA and Huiskamp R (1989) Active uptake and extravesicular storage of *m*-iodobenzylguanidine in human neuroblastoma SK-N-SH cells. *Cancer Res* **49**: 2941–2944

Strickland DK, Vaidyanathan G and Zalutsky MR (1994) Cytotoxicity of alpha-particle-emitting *m*-[$^{211}$At]astatobenzylguanidine on human neuroblastoma cells. *Cancer Res* **54**: 5414–5419

© Cancer Research Campaign 1997

Vaidynathan G and Zalutsky MR (1992) 1-(*m*-[$^{211}$At]Astatobenzyl)guanidine: Synthesis via astato demetalation and preliminary in vitro and in vivo evaluation. *Bioconjugate Chem* **3**: 499–503

Vaidyanathan G and Zalutsky MR (1993) No-carrier-added synthesis of *meta*-[$^{131}$I]-iodobenzylguanidine. *Appl Radiat Isot* **44**: 621–628

Vaidyanathan G, Strickland DK and Zalutsky MR (1994*a*) *Meta*-[$^{211}$At]astatobenzylguanidine: Further evaluation of a potential therapeutic agent. *Int J Cancer* **57**: 908–913

Vaidyanathan G, Affleck DJ and Zalutsky MR (1994*b*) 4-[$^{18}$F]Fluoro-3-iodobenzylguanidine: A potential MIBG analog for positron emission tomography. *J Med Chem* **37**: 3655–3662

Vaidyanathan G, Affleck DJ and Zalutsky MR (1995) Validation of 4-[$^{18}$F]fluoro-3-iodobenzylguanidine as a potential PET analog of MIBG. *J Nucl Med* **36**: 644–650

Vaidyanathan G, Affleck DJ and Zalutsky MR (1996) No-carrier-added 4-fluoro-3-[$^{131}$I]iodobenzylguanidine and 3-[$^{211}$At]astato-4-fluorobenzylguanidine. *Bioconjugate Chem* **7**: 102–107

Vaidyanathan G, Zhao X-G, Strickland DK and Zalutsky MR (1997) No-carrier-added 4-fluoro-3-[$^{131}$I]iodobenzylguanidine: An MIBG analogue with improved binding to neuroblastoma cells in vitro and myocardial retention in vivo. *J Nucl Med* 38: 330–334

Valette H, Loc'h C, Mardon K, Bendriem B, Merlet P, Fuseau C, Sabry S, Raffel D, Mazière B and Syrota A (1993) Bromine-76-metabromobenzylguanidine: A PET radiotracer for mapping sympathetic nerves of the heart. *J Nucl Med* **34**: 1739–1744

Zalutsky MR, Garg PK, Friedman HS and Bigner DD (1989) Labeling monoclonal antibodies and $F(ab')_2$ fragments with the alpha particle emitting nuclide astatine-211: Preservation of immunoreactivity and in vivo localizing capacity. *Proc Natl Acad Sci USA* **86**: 7149–7153

© Cancer Research Campaign 1997


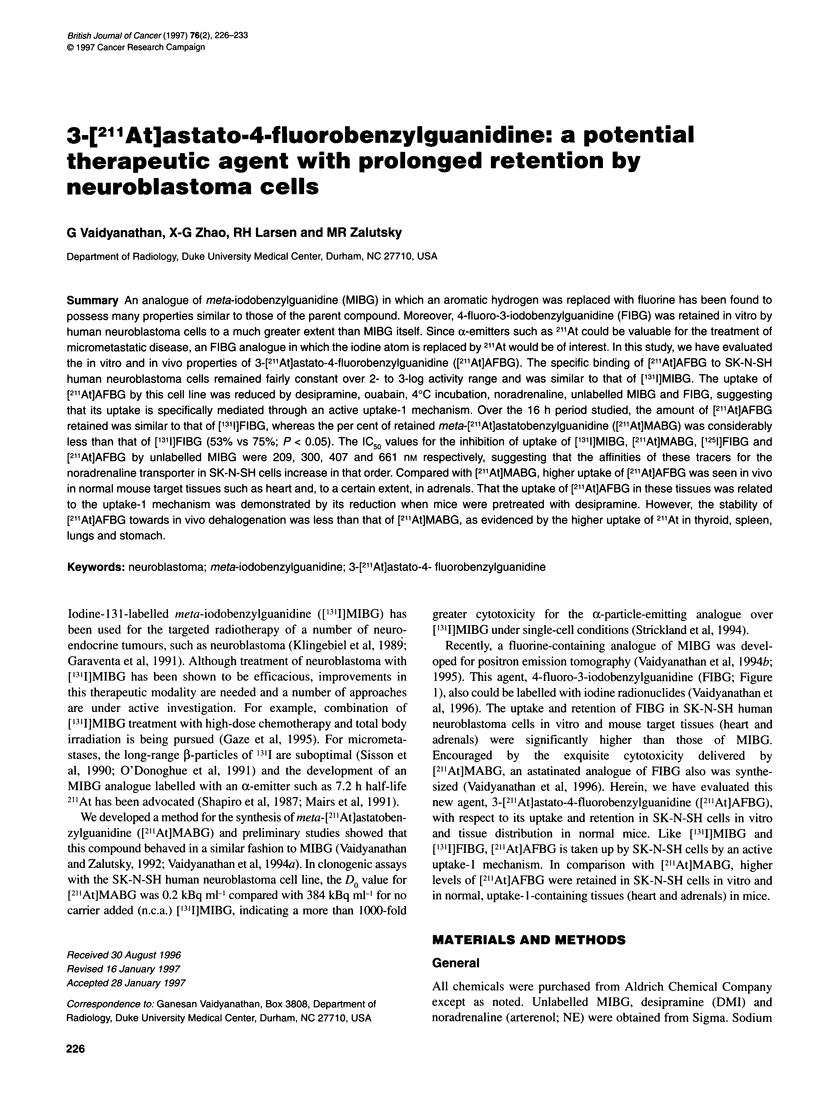

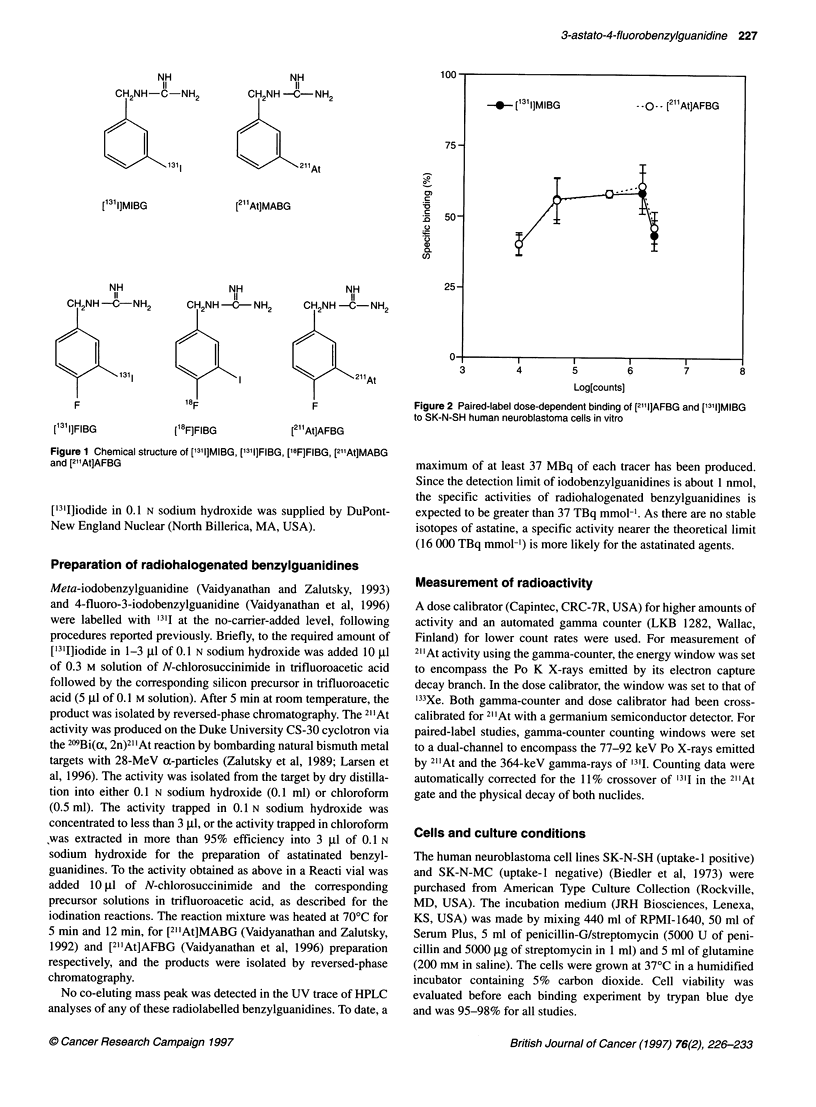

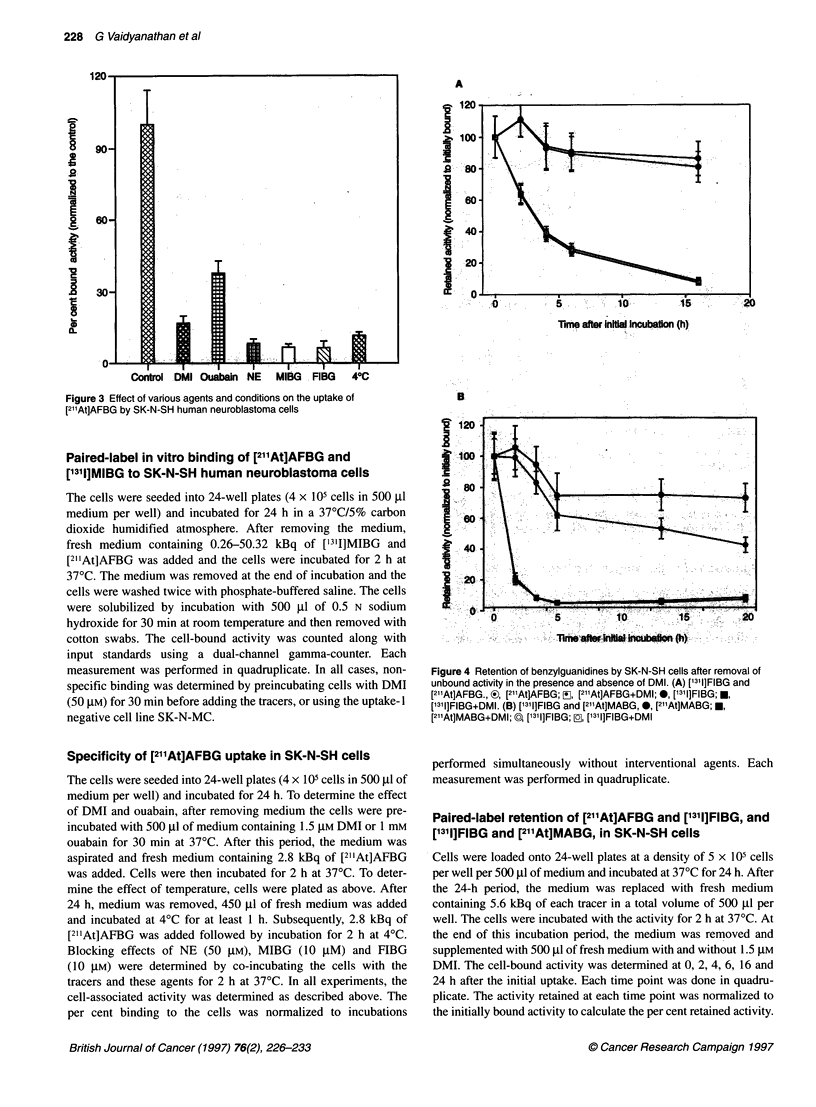

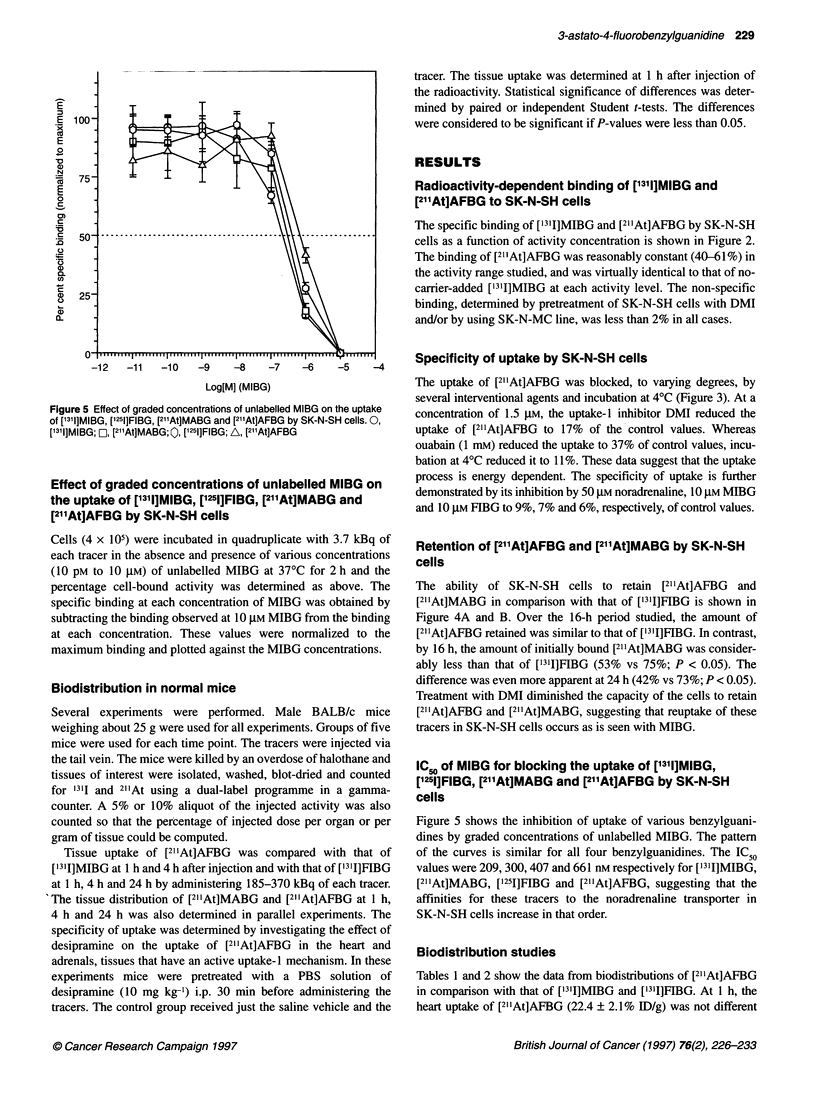

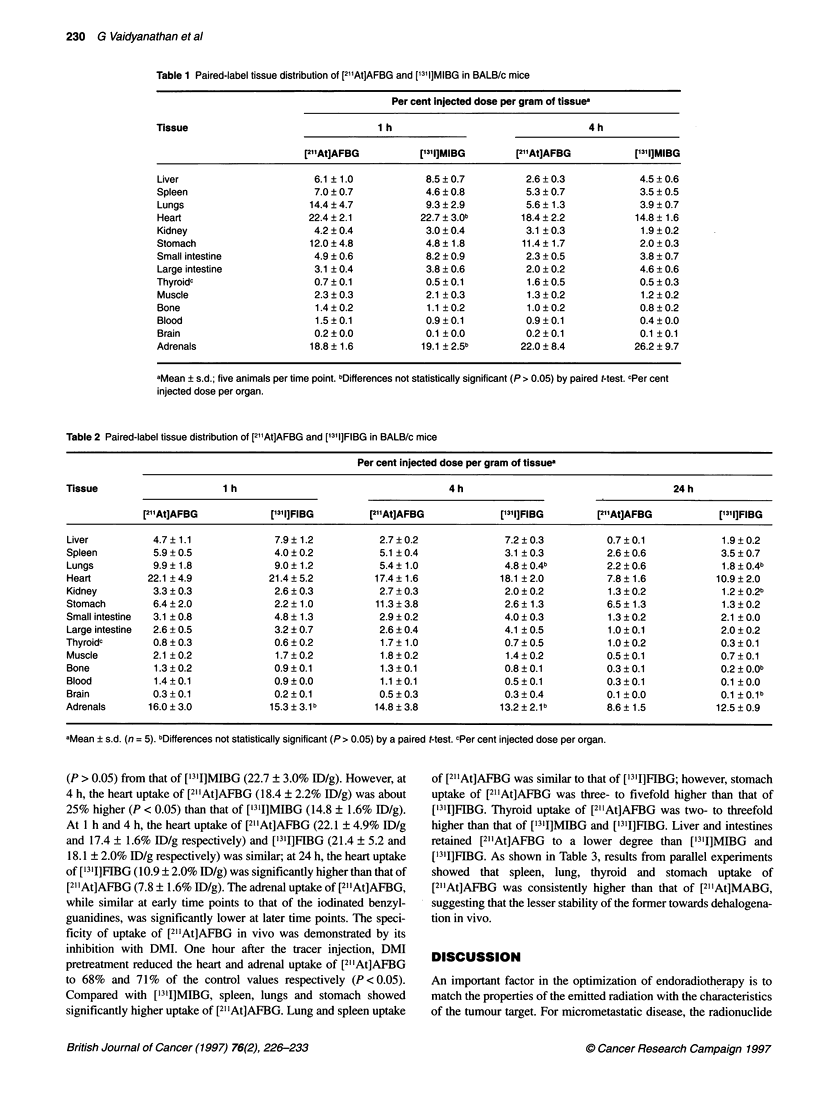

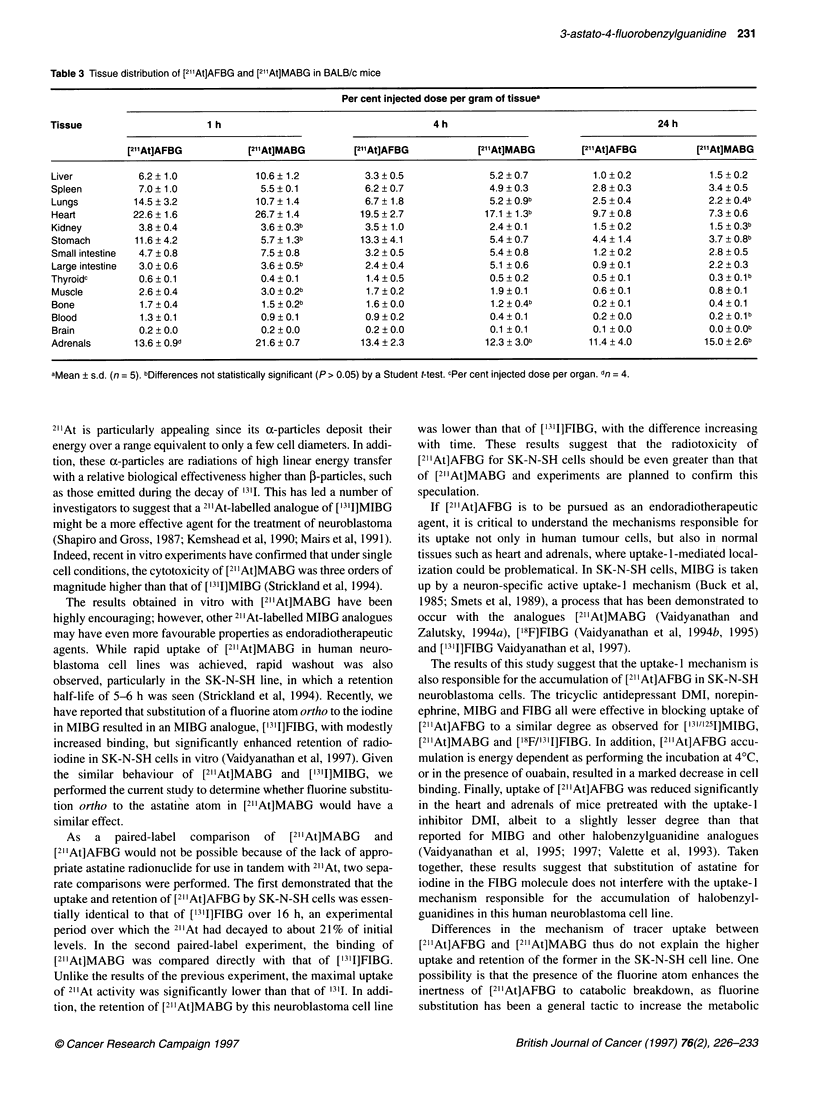

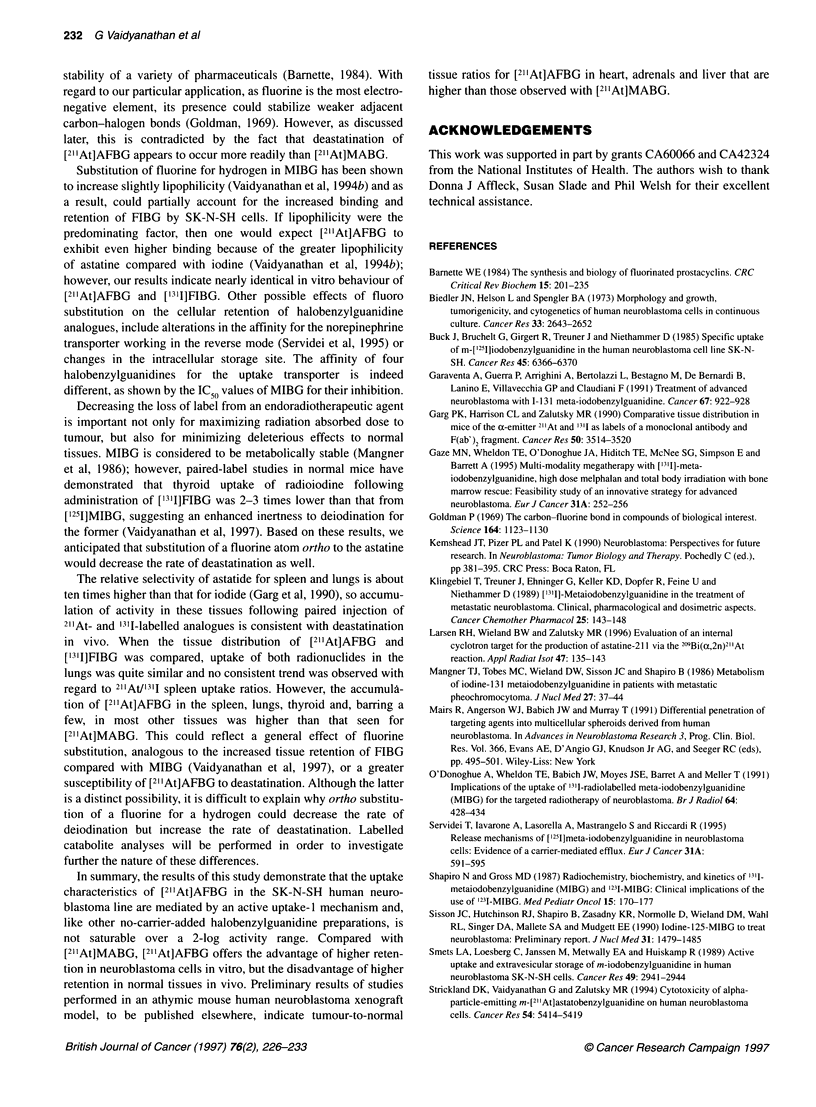

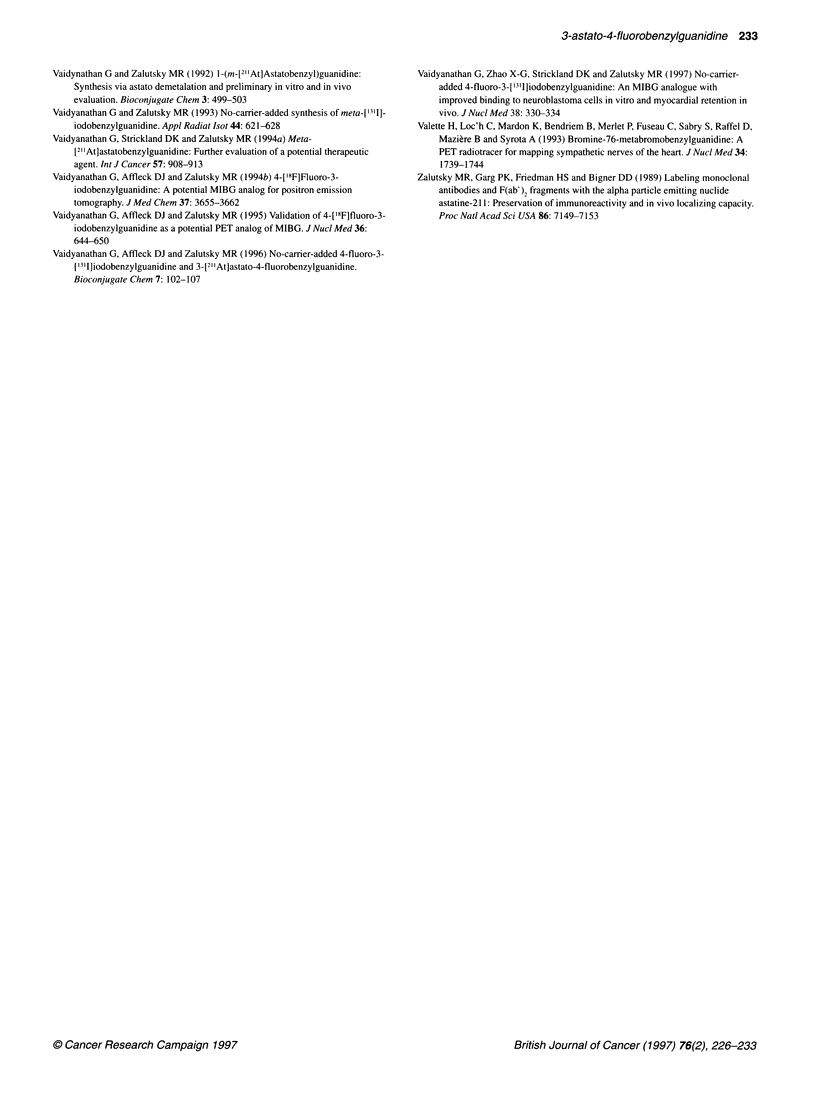


## References

[OCR_00799] Barnette W. E. (1984). The synthesis and biology of fluorinated prostacyclins.. CRC Crit Rev Biochem.

[OCR_00803] Biedler J. L., Helson L., Spengler B. A. (1973). Morphology and growth, tumorigenicity, and cytogenetics of human neuroblastoma cells in continuous culture.. Cancer Res.

[OCR_00808] Buck J., Bruchelt G., Girgert R., Treuner J., Niethammer D. (1985). Specific uptake of m-[125I]iodobenzylguanidine in the human neuroblastoma cell line SK-N-SH.. Cancer Res.

[OCR_00813] Garaventa A., Guerra P., Arrighini A., Bertolazzi L., Bestagno M., De Bernardi B., Lanino E., Villavecchia G. P., Claudiani F. (1991). Treatment of advanced neuroblastoma with I-131 meta-iodobenzylguanidine.. Cancer.

[OCR_00818] Garg P. K., Harrison C. L., Zalutsky M. R. (1990). Comparative tissue distribution in mice of the alpha-emitter 211At and 131I as labels of a monoclonal antibody and F(ab')2 fragment.. Cancer Res.

[OCR_00823] Gaze M. N., Wheldon T. E., O'Donoghue J. A., Hilditch T. E., McNee S. G., Simpson E., Barrett A. (1995). Multi-modality megatherapy with [131I]meta-iodobenzylguanidine, high dose melphalan and total body irradiation with bone marrow rescue: feasibility study of a new strategy for advanced neuroblastoma.. Eur J Cancer.

[OCR_00831] Goldman P. (1969). The carbon-fluorine bond in compounds of biological interest.. Science.

[OCR_00840] Klingebiel T., Treuner J., Ehninger G., Keller K. D., Dopfer R., Feine U., Niethammer D. (1989). [131I]-metaiodobenzylguanidine in the treatment of metastatic neuroblastoma. Clinical, pharmacological and dosimetric aspects.. Cancer Chemother Pharmacol.

[OCR_00847] Larsen R. H., Wieland B. W., Zalutsky M. R. (1996). Evaluation of an internal cyclotron target for the production of 211At via the 209Bi (alpha,2n)211 at reaction.. Appl Radiat Isot.

[OCR_00857] Mairs R. J., Angerson W. J., Babich J. W., Murray T. (1991). Differential penetration of targeting agents into multicellular spheroids derived from human neuroblastoma.. Prog Clin Biol Res.

[OCR_00852] Mangner T. J., Tobes M. C., Wieland D. W., Sisson J. C., Shapiro B. (1986). Metabolism of iodine-131 metaiodobenzylguanidine in patients with metastatic pheochromocytoma.. J Nucl Med.

[OCR_00866] O'Donoghue J. A., Wheldon T. E., Babich J. W., Moyes J. S., Barrett A., Meller S. T. (1991). Implications of the uptake of 131I-radiolabelled meta-iodobenzylguanidine (mIBG) for the targeted radiotherapy of neuroblastoma.. Br J Radiol.

[OCR_00872] Servidei T., Iavarone A., Lasorella A., Mastrangelo S., Riccardi R. (1995). Release mechanisms of [125I]meta-iodobenzylguanidine in neuroblastoma cells: evidence of a carrier-mediated efflux.. Eur J Cancer.

[OCR_00878] Shapiro B., Gross M. D. (1987). Radiochemistry, biochemistry, and kinetics of 131I-metaiodobenzylguanidine (MIBG) and 123I-MIBG: clinical implications of the use of 123I-MIBG.. Med Pediatr Oncol.

[OCR_00883] Sisson J. C., Hutchinson R. J., Shapiro B., Zasadny K. R., Normolle D., Wieland D. M., Wahl R. L., Singer D. A., Mallette S. A., Mudgett E. E. (1990). Iodine-125-MIBG to treat neuroblastoma: preliminary report.. J Nucl Med.

[OCR_00888] Smets L. A., Loesberg C., Janssen M., Metwally E. A., Huiskamp R. (1989). Active uptake and extravesicular storage of m-iodobenzylguanidine in human neuroblastoma SK-N-SH cells.. Cancer Res.

[OCR_00893] Strickland D. K., Vaidyanathan G., Zalutsky M. R. (1994). Cytotoxicity of alpha-particle-emitting m-[211At]astatobenzylguanidine on human neuroblastoma cells.. Cancer Res.

[OCR_00916] Vaidyanathan G., Affleck D. J., Zalutsky M. R. (1994). (4-[18F]fluoro-3-iodobenzyl)guanidine, a potential MIBG analogue for positron emission tomography.. J Med Chem.

[OCR_00926] Vaidyanathan G., Affleck D. J., Zalutsky M. R. (1996). No-carrier-added (4-fluoro-3-[131I]iodobenzyl)guanidine and (3-[211At]astato-4-fluorobenzyl)guanidine.. Bioconjug Chem.

[OCR_00921] Vaidyanathan G., Affleck D. J., Zalutsky M. R. (1995). Validation of 4-[fluorine-18]fluoro-3-iodobenzylguanidine as a positron-emitting analog of MIBG.. J Nucl Med.

[OCR_00911] Vaidyanathan G., Strickland D. K., Zalutsky M. R. (1994). Meta-[211At]astatobenzylguanidine: further evaluation of a potential therapeutic agent.. Int J Cancer.

[OCR_00902] Vaidyanathan G., Zalutsky M. R. (1992). 1-(m-[211At]astatobenzyl)guanidine: synthesis via astato demetalation and preliminary in vitro and in vivo evaluation.. Bioconjug Chem.

[OCR_00907] Vaidyanathan G., Zalutsky M. R. (1993). No-carrier-added synthesis of meta-[131I]iodobenzylguanidine.. Appl Radiat Isot.

[OCR_00931] Vaidyanathan G., Zhao X. G., Strickland D. K., Zalutsky M. R. (1997). No-carrier-added iodine-131-FIBG: evaluation of an MIBG analog.. J Nucl Med.

[OCR_00940] Valette H., Loc'h C., Mardon K., Bendriem B., Merlet P., Fuseau C., Sabry S., Raffel D., Mazière B., Syrota A. (1993). Bromine-76-metabromobenzylguanidine: a PET radiotracer for mapping sympathetic nerves of the heart.. J Nucl Med.

[OCR_00944] Zalutsky M. R., Garg P. K., Friedman H. S., Bigner D. D. (1989). Labeling monoclonal antibodies and F(ab')2 fragments with the alpha-particle-emitting nuclide astatine-211: preservation of immunoreactivity and in vivo localizing capacity.. Proc Natl Acad Sci U S A.

